# Supplementary figures and images for: Full-length transcriptome revealed the accumulation of polyunsaturated fatty acids in developing seeds of Plukenetia volubilis
Source: PeerJ. 2022 Sep 20;10:e13998. doi: 10.7717/peerj.13998 (PMC9504451; doi:10.7717/peerj.13998)

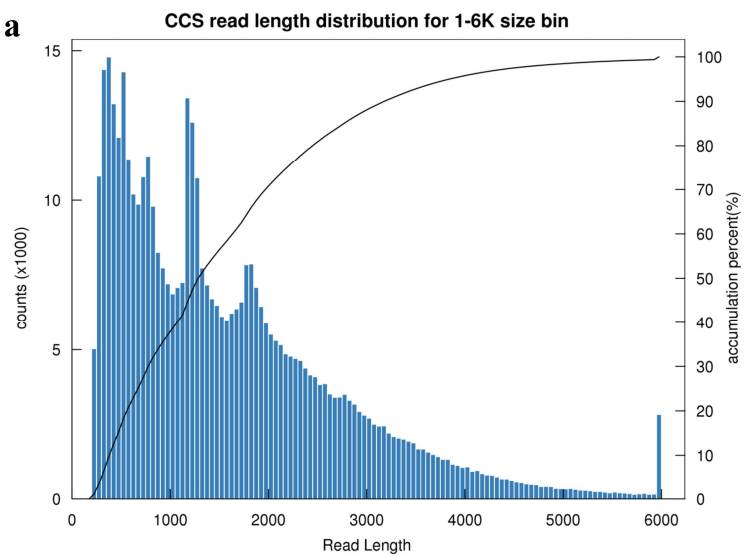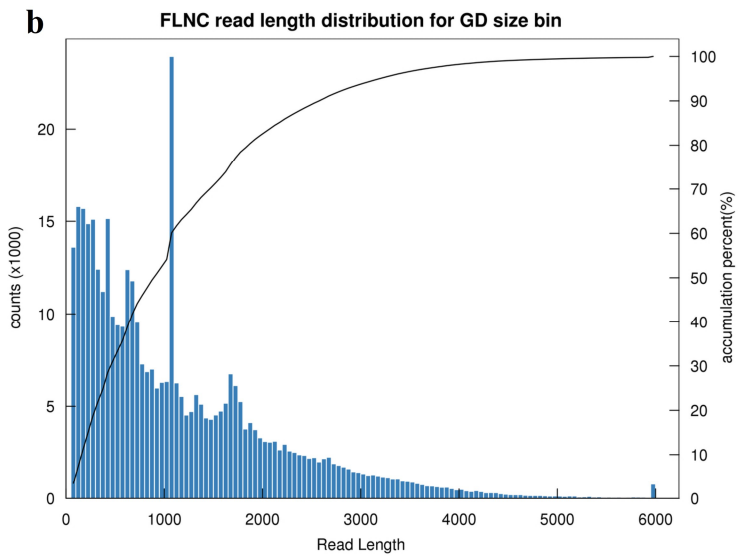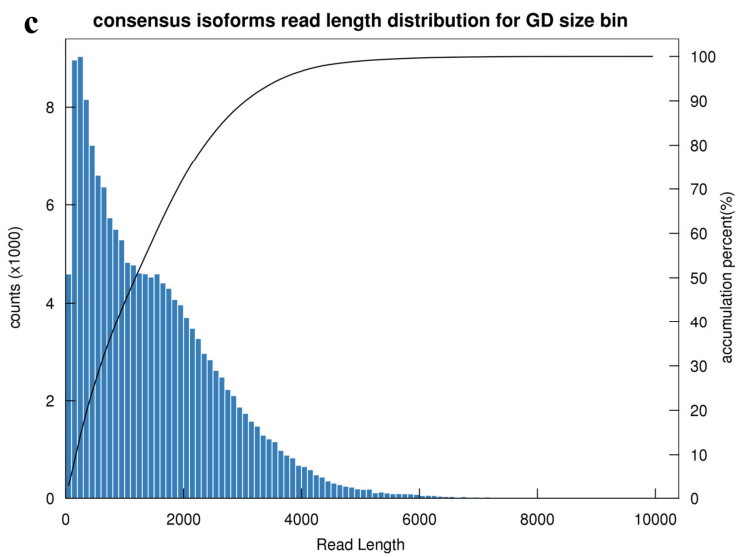

Supplement: Figure S1 — (a) Length and nu mber distribution of CCS. (b) Length Numbe r and number distribution of FLNC reads. (c) Length and number distribution of corrected isoforms. [file peerj-10-13998-s002.pdf]

a

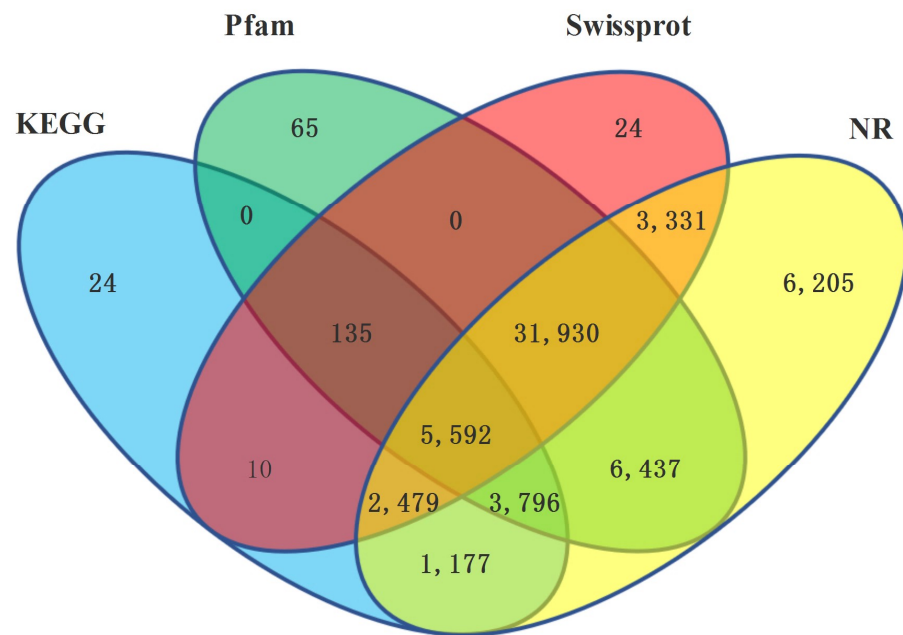

b

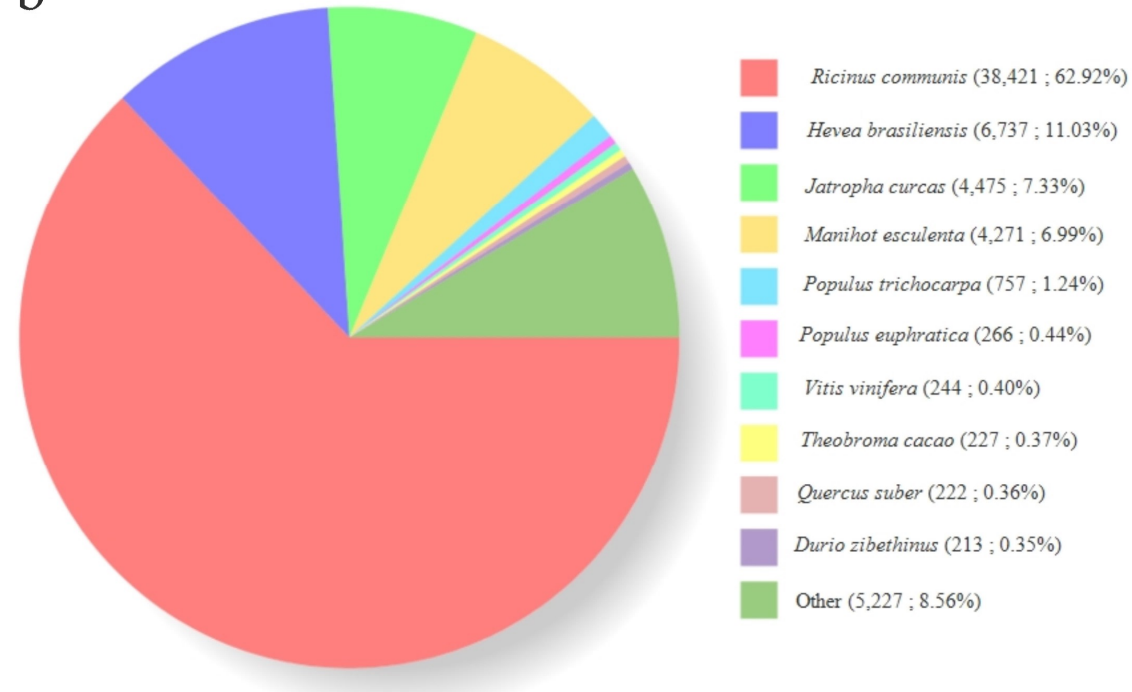

Supplement: Figure S2 — (a) The distribution of annotated unigenes in different databases. (b) Species distribution of annotated unigenes in Nr database. [file peerj-10-13998-s003.pdf]

**a**

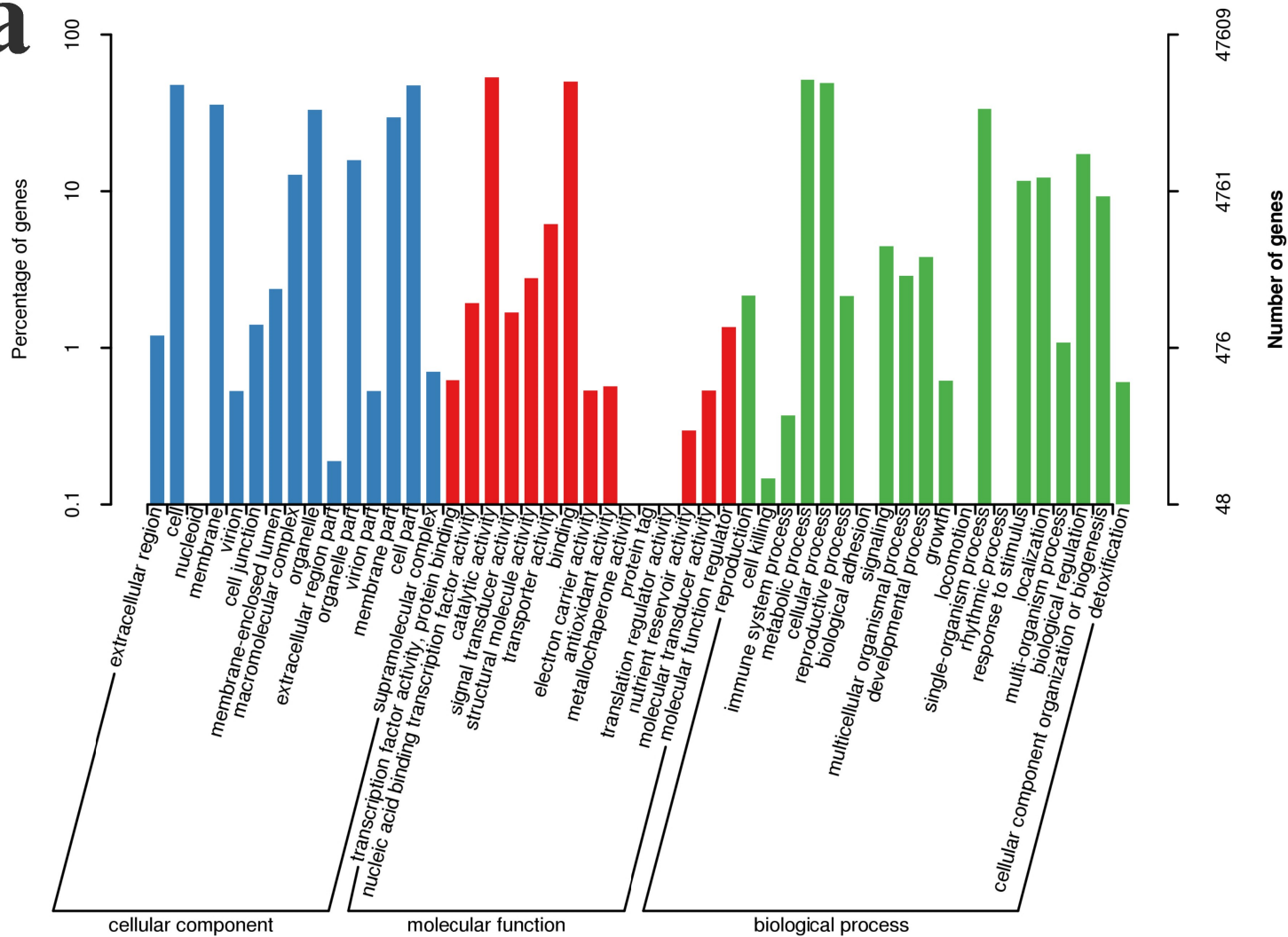

**b**

### COG Function Classification of Consensus Sequence

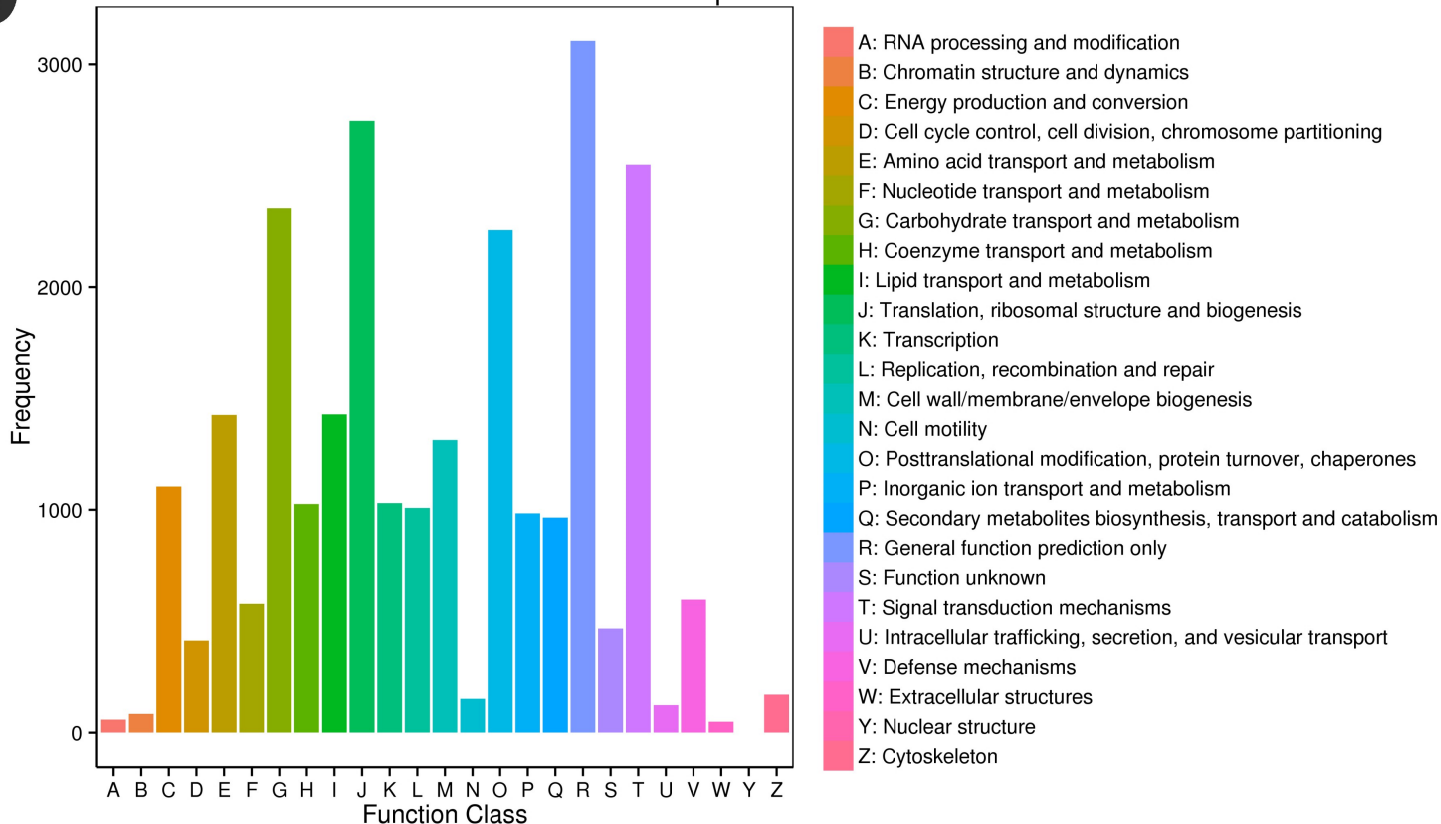

Supplement: Figure S3 [file peerj-10-13998-s004.pdf]

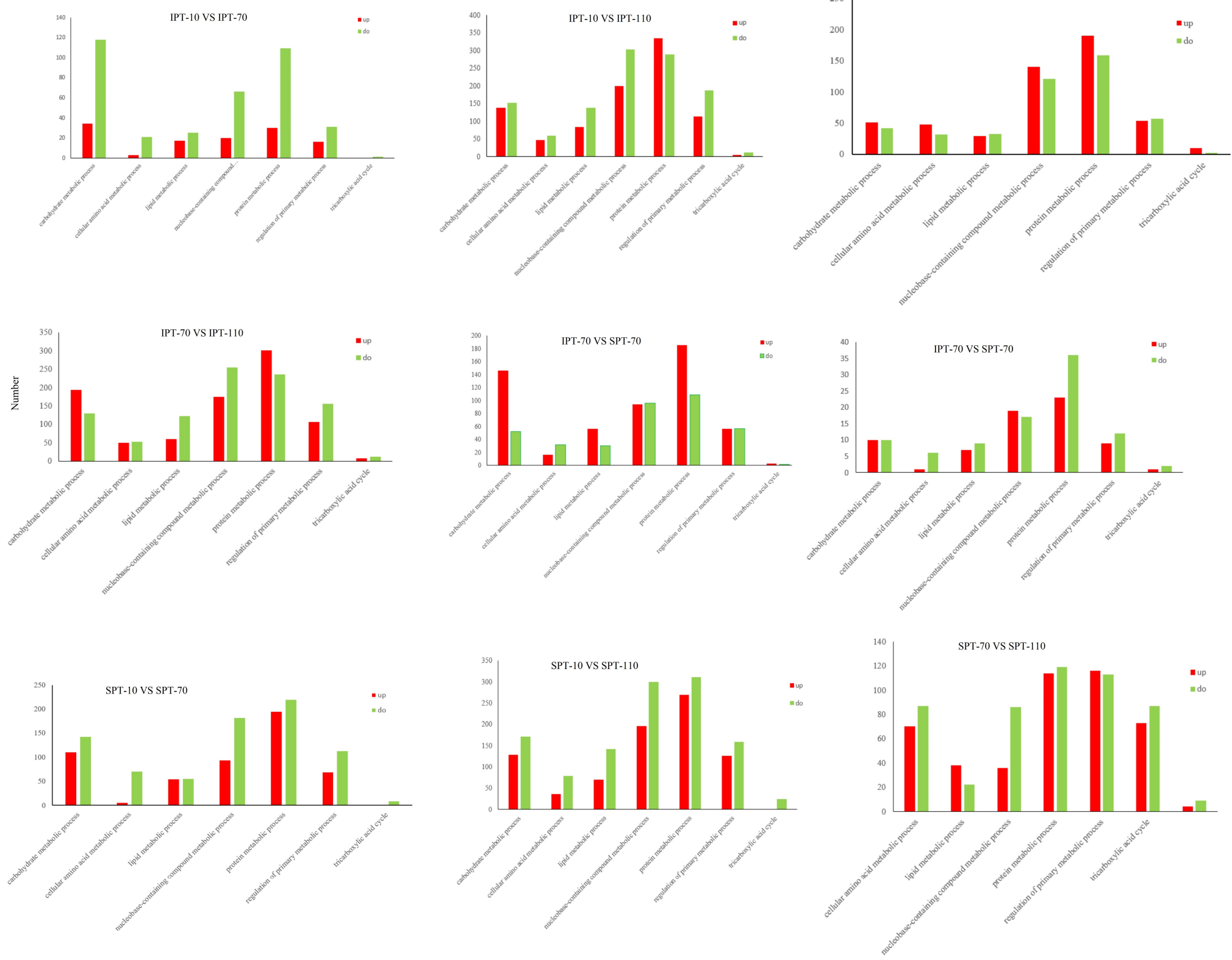

Supplement: Figure S4 [file peerj-10-13998-s005.pdf]

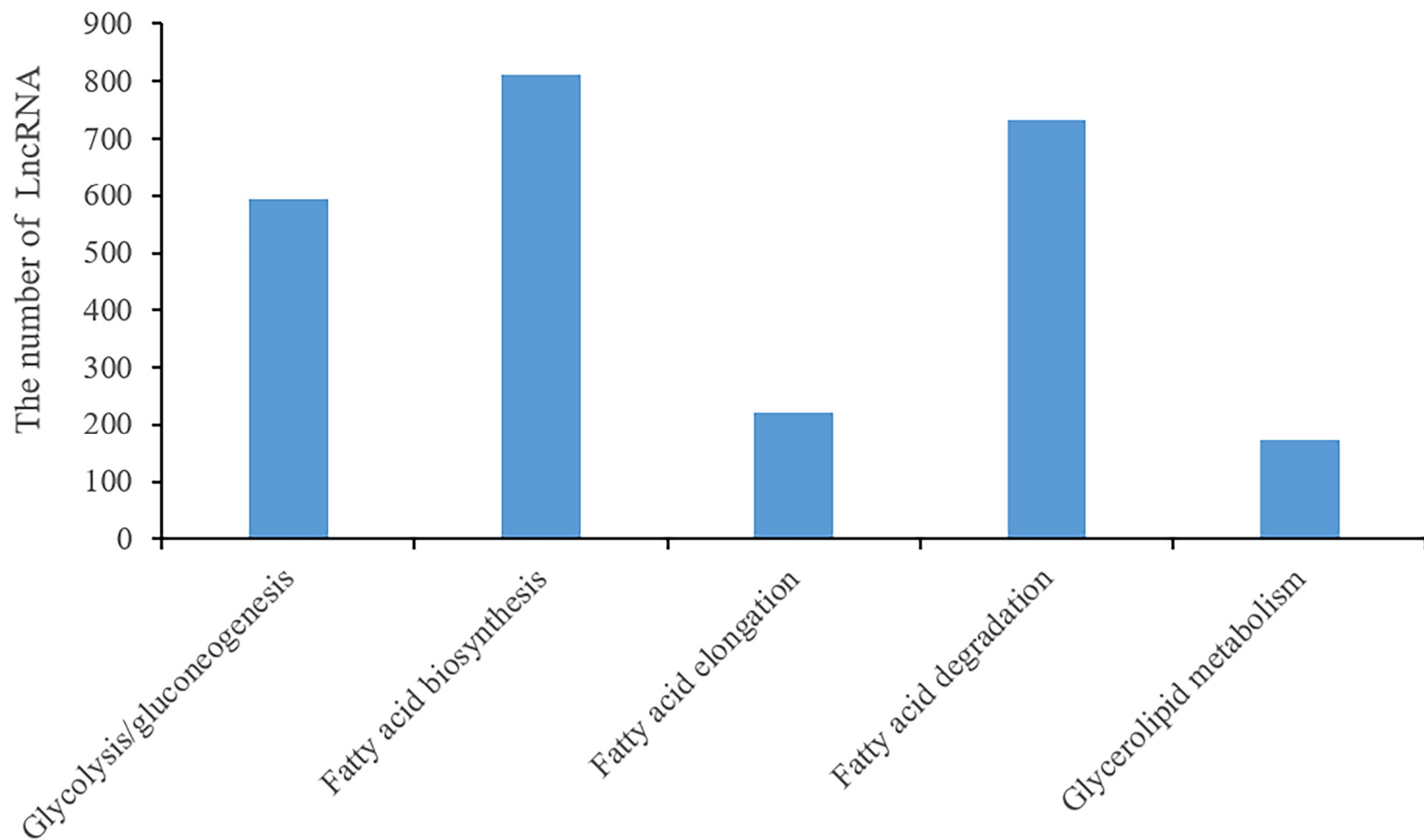

Supplement: Figure S5 [file peerj-10-13998-s006.pdf]
